# Mutations of two amino acids in VP5 mediate the attenuation of human rotavirus vaccine: evidence from *in vitro* and *in vivo* studies

**DOI:** 10.1128/jvi.01067-25

**Published:** 2025-10-08

**Authors:** Theresa K. Bessey, Yuhuan Wang, Sung-Sil Moon, Liliana Sanchez-Tacuba, Philippe H. Jaïs, Harry B. Greenberg, Baoming Jiang

**Affiliations:** 1 Division of Viral Diseases, Centers for Disease Control and Prevention (CDC) 1242, Atlanta, Georgia, USA; 2 Department of Medicine and Microbiology and Immunology, Stanford University School of Medicine 10624 , Stanford, California, USA; 3 Eukarÿs SAS 637109 , Evry-Courcouronnes, France; University of Michigan Medical School, Ann Arbor, Michigan, USA

**Keywords:** *in vivo*, *in vitro*, mutations, vaccine, rotavirus

## Abstract

Various vaccines, like polio, measles, and rotavirus, have been developed by serial-passaging in cell culture. Live oral rotavirus vaccines have been shown to be generally safe, but mechanisms of attenuation are not known. We have used a new, entirely plasmid-based reverse genetics system to artificially generate the novel human rotavirus vaccine strain CDC-9 (G1P[8]) and analyze the effect of the mutations within the VP4 gene on adaptation *in vitro* and attenuation *in vivo*. We demonstrated that out of the 6 amino acid mutations that appeared after serial passaging in Vero cells, mutations of wild-type CDC-9 P11 at VP4 AA331 and AA385 each or in combination were associated with increased replication *in vitro* comparable to cell-culture adapted CDC-9 P45. Neonatal rats infected with the single AA331 or AA385 mutant had reduced viral shedding, comparable to cell-culture passaged CDC-9 P45. We observed additional reduced shedding in neonatal rats that were infected with combination mutants harboring mutations at position AA331_385_388, indicative of a slight additive effect. Our data indicate that mutations in the VP5* region of the VP4 gene, particularly at position AA331 and AA385, are the determining factor for *in vitro* replication adaptation and *in vivo* attenuation of a G1P[8] rotavirus vaccine. This information provides great potential for targeted mutation in rotavirus vaccine generation instead of labor-consuming serial passaging in cell culture.

**IMPORTANCE:**

Live oral rotavirus vaccines have been developed through serial passaging in cell culture and found to be generally safe and efficacious in children. Live vaccines are also found to be associated with rare but severe adverse events, such as intussusception, in vaccinated children. Mechanisms for vaccine attenuation and adverse effects are unknown. We have developed a novel human rotavirus vaccine strain (CDC-9) and demonstrated several amino acid mutations in the VP4 gene of cell-passaged virus. In the present study, we identified two key amino acid mutations via reverse genetics technology in VP4 that mediated enhanced growth in cell culture, including a human intestinal cell line, reduced virus shedding, and downregulated inflammatory response in neonatal rats. This study is the first to identify the molecular signatures that define attenuation of human rotavirus vaccine and should help provide guidance for developing new generations of safe and effective vaccines.

## INTRODUCTION

Serial-passaging in cell culture has been used to generate attenuated virus strains for measles, polio, mumps, rubella, varicella, and rotavirus vaccine development (1–6). Sequence changes within the genome can be correlated with viral adaptation in tissue culture and attenuation *in vivo* making serial-passaging an important tool for the generation of live-attenuated vaccine strains. Multiple licensed rotavirus vaccines, including human rotavirus vaccines Rotarix (G1P[8]) and Rotavac (G9P[11]), have been developed by serial passaging of wild-type rotavirus isolates in cell culture, found to be attenuated and immunogenic in children, and introduced into routine immunization in more than 120 countries (6–8). However, rotavirus infection still is the leading cause of severe gastroenteritis and results in the death of around 128,000 children under the age of 5 worldwide per year (9). Live oral rotavirus vaccines are generally safe but are associated with a rare but severe adverse event, intussusception (10–12), and require constant surveillance. Therefore, an understanding of the genetic basis of rotavirus attenuation is crucial to develop new vaccines with an improved safety profile. Rotarix ancestor strain 89-12 (G1P[8]) was passaged for 26 times in primary African Green monkey kidney (AGMK) cells followed by 7 passages in Vero cell line. Sequence analysis of attenuated Rotarix and ancestor strain 89-12 revealed a total of 5 amino acid (AA) mutations in VP4 (13). Inoculation of infants with Rotarix resulted in reduced shedding and no diarrhea compared to infection with the 89-12 parental strain (6). Despite overwhelming safety and efficacy data, the genetic basis and mechanism for attenuation and safety of these vaccines remain unknown.

Rotavirus belongs to the family of Sedoreoviridae and is a non-enveloped, dsRNA virus. The genome is comprised of 11 segments encoding 6 structural proteins (VP1-VP4, VP6-VP7) and 6 non-structural proteins (NSP1-NSP6) (14). The outer layer of the triple-layered, infectious rotavirus particle is comprised of structural proteins VP7 and VP4. VP4 is responsible for cell attachment and entry and is proteolytically cleaved into two functional subunits, VP8* and VP5*, for infection (15). Various studies have investigated the functionality of a VP8* subunit vaccine to prevent rotavirus infection in children despite its variable epitopes (16–19). By contrast, VP5* is more conserved in sequence among human strains, essential for cell membrane permeabilization and cell entry upon rotavirus infection (20), and will be analyzed in this study to understand its effect on adaptation for growth in cells and attenuation in animals.

We have developed a novel human rotavirus vaccine strain CDC-9 (G1P[8]) for live-attenuated or inactivated human rotavirus vaccination. CDC-9 was isolated from fecal specimen of an infected child in the USA and was serially passaged 7 times in MA104 cells (P7) followed by 38 passages in Vero cells for a total of 45 passages (P45) (21). CDC-9 P7 was also passaged 5 more times in MA104 cells for a total of 12 passages (P12) for use in this study. Since there were no amino acid sequence changes from stool to passage 12 in MA104 cells, CDC-9 P12 was considered a wild-type virus. During serial passaging up to passage (P)28 in Vero cells, we observed 5 AA changes in VP4, and one each in VP6, NSP1, and NSP5 compared to stool and exclusively MA104-passaged CDC-9 P11/12. When we continued serial passaging CDC-9 up to P45, we noted one AA change in VP1, a 10 AA deletion in VP2, as well as one additional AA mutation in VP4 at position AA499 (22). We showed that CDC-9 P45 and CDC-9 P28 grew to higher titers in various cell lines and were associated with reduced shedding in neonatal rats compared to infection with CDC-9 P11/12, demonstrating that indeed, serial passaging of CDC-9 led to adaptation and attenuation (22). Interestingly, CDC-9 P28 and CDC-9 P45 replicated to comparable high titers *in vitro* independent of the VP2 deletion occurring between P28 and P45. This indicates that VP4 mutations are the driving factor of cell culture adaptation during serial passages. Of the six amino acid mutations observed in CDC-9 VP4, none occurred in VP8* and four in the membrane fusion domain of VP5*, suggesting mutations in VP5* could be responsible for the attenuation and adaptation of CDC-9.

Recent studies have used an entirely plasmid-based reverse genetics system to generate reassortant rotavirus strains with human VP4 gene on a simian (SA11) backbone (23–25). These studies paved the way for the growth of P[4] and P[8] clinical isolates and for better understanding the adaptation of human rotavirus strains in cell culture. However, to date, no studies have identified the specific AA sequence at specific locations within rotavirus genes that are responsible for the adaptation and attenuation of human rotavirus vaccine strains. In the present study, we took advantage of complete passage history and fully delineated nucleotide and amino acid sequences to examine associations between specific mutations in VP5* and documented phenotypes *in vitro* and *in vivo* of the human rotavirus vaccine strain CDC-9 utilizing reverse genetics technology. The findings from the present study would help expedite the development, skipping the time-consuming process of serial passaging in cell culture, and improve the safety of rotavirus vaccines.

## RESULTS

### Importance of rotavirus VP4 for infectivity *in vitro* and virulence *in vivo*

**Fig 1:**
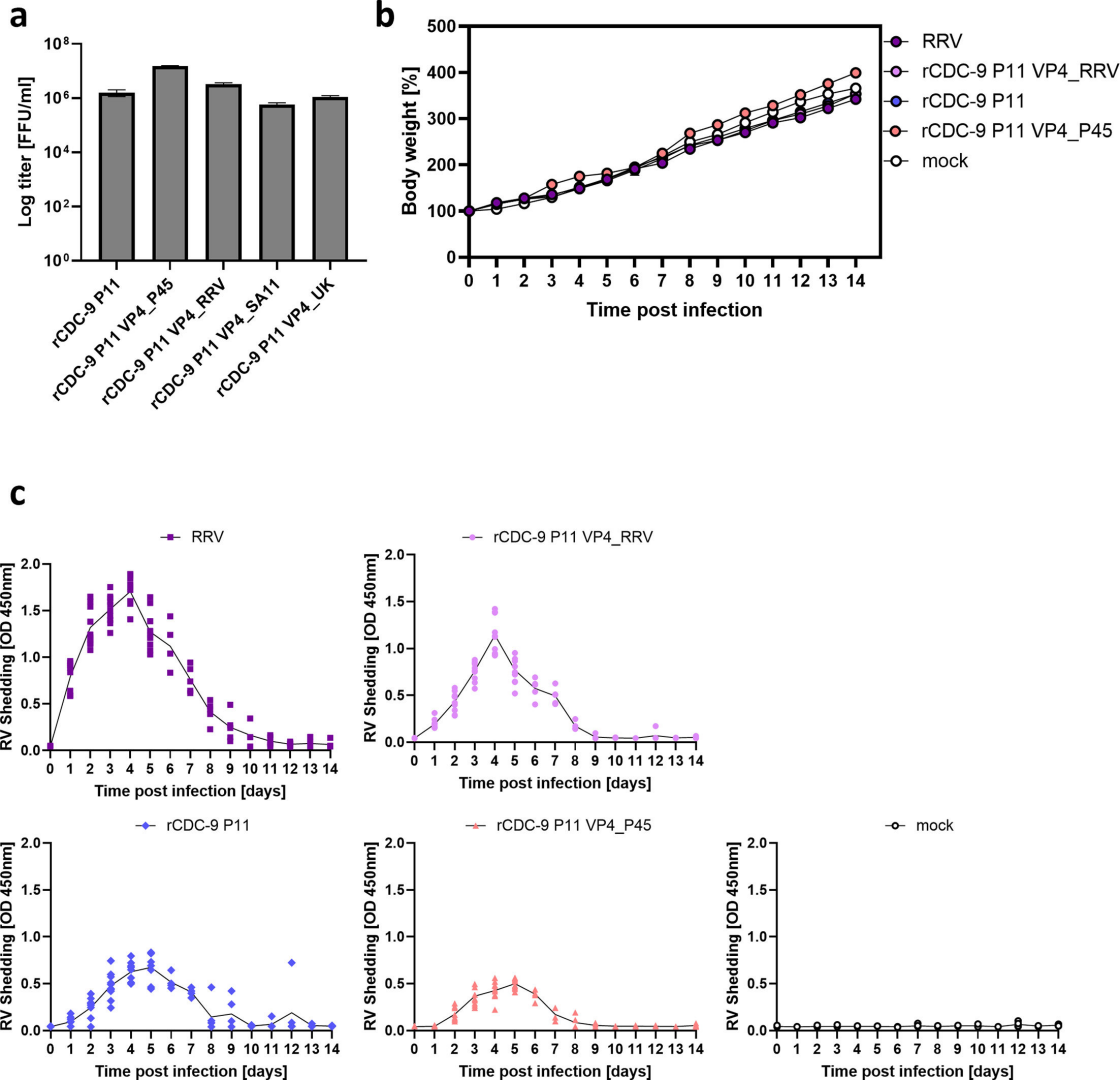
Effect of VP4 of human and simian origin on rotavirus replication *in vitro* and shedding *in vivo*. (**a**) Titer of recombinant VP4 mono-reassortments on CDC-9 P11 background in MA104 cells. *N* = 6. One-way ANOVA was performed comparing mean titers of recombinant strains to rCDC-9 P11. (**b**) Infection of 5-day-old neonatal rats with 1 × 10^7^ FFU of the indicated recombinant rotavirus strains. Bodyweight was monitored for 14 days post infection. *N* = 10 (days 0–5) and *N* = 5 (days 6–14). (**c**) Shedding in neonatal rats after infection with 1 × 10^7^ FFU of the indicated recombinant rotavirus strains. Shedding was measured in rectal swabs by Premier Rotaclone EIA. Two-way ANOVA with multiple test comparison was performed to compare mean shedding after infection with recombinant strains to mean shedding after rCDC-9 P11 infection. ns, not significant (*P* ≥ 0.05); *P < 0.05. Shown are mean and SEM for all experiments.

Rotavirus VP4 protein is essential for cell attachment, cell wall penetration, and entry into the cytoplasm. To understand the effect of VP4 on infectivity and pathogenicity, we generated recombinant mono-reassortment strains on a CDC-9 P11 background with different VP4 genes from simian (SA11), bovine (UK), and human (CDC-9) rotaviruses (rCDC-9 P11 VP4_RRV, rCDC-9 P11 VP4_SA11, rCDC-9 P11 VP4_UK, and rCDC-9 P11 VP4_P45) (Fig. 1a). A recombinant mono-reassortment between wild-type CDC-9 P11 with only the VP4 gene from attenuated CDC-9 P45 (rCDC-9 P11 VP4_P45) demonstrated a significant increase in titer in MA104 cells when compared with rCDC-9 P11, indicating that VP4 in CDC-9 P45 is primarily responsible for the enhanced cell culture replication capacity of this strain. Interestingly, the titers of mono-reassortment strains with simian (RRV or SA11) or bovine (UK) VP4 remained lower than rCDC-9 P11 VP4_P45 most likely due to the attenuated nature of CDC-9 P45 VP4. To determine the effect of VP4 origin on *in vivo* infectivity and pathogenesis, we initially infected 5-day-old neonatal rats with 1 × 10^7^ FFU of rhesus rotavirus (RRV), rCDC-9 P11, rCDC-9 P11 harboring RRV VP4 (rCDC-9 P11 VP4_RRV), rCDC-9 P11 harboring P45 VP4 (rCDC-9 P11 VP4_P45), or mock control. Body weight in neonatal rats after virus infection reflected the general state and health of these animals (Fig. 1b). We observed comparable body weight gain in animals infected with rCDC-9 P11 VP4_P45 and mock control but reduced body weight gain in animals infected with RRV and rCDC-9 P11 VP4_RRV, and rCDC-9 P11. Of note, no diarrhea was observed in any infected animals. We observed that animals infected with RRV had the highest level of rotavirus shedding, followed by rCDC-9 P11 VP4_RRV on day 4 (Fig. 1c). Rats with rCDC-9 P11 infection had moderate levels of shedding but significantly reduced rotavirus shedding after infection with rCDC-9 P11 VP4_P45 (Fig. 1c). These results indicate that the VP4 gene of the attenuated vaccine strain CDC-9 P45 mediates reduced shedding in neonatal rats and the increased virus growth in cell culture and demonstrate that this vaccine strain is adapted *in vitro* and attenuated *in vivo* due to sequence changes within the VP4 gene.

### Generation of recombinant CDC-9 strains with mutations in VP4 gene

**Fig 2:**
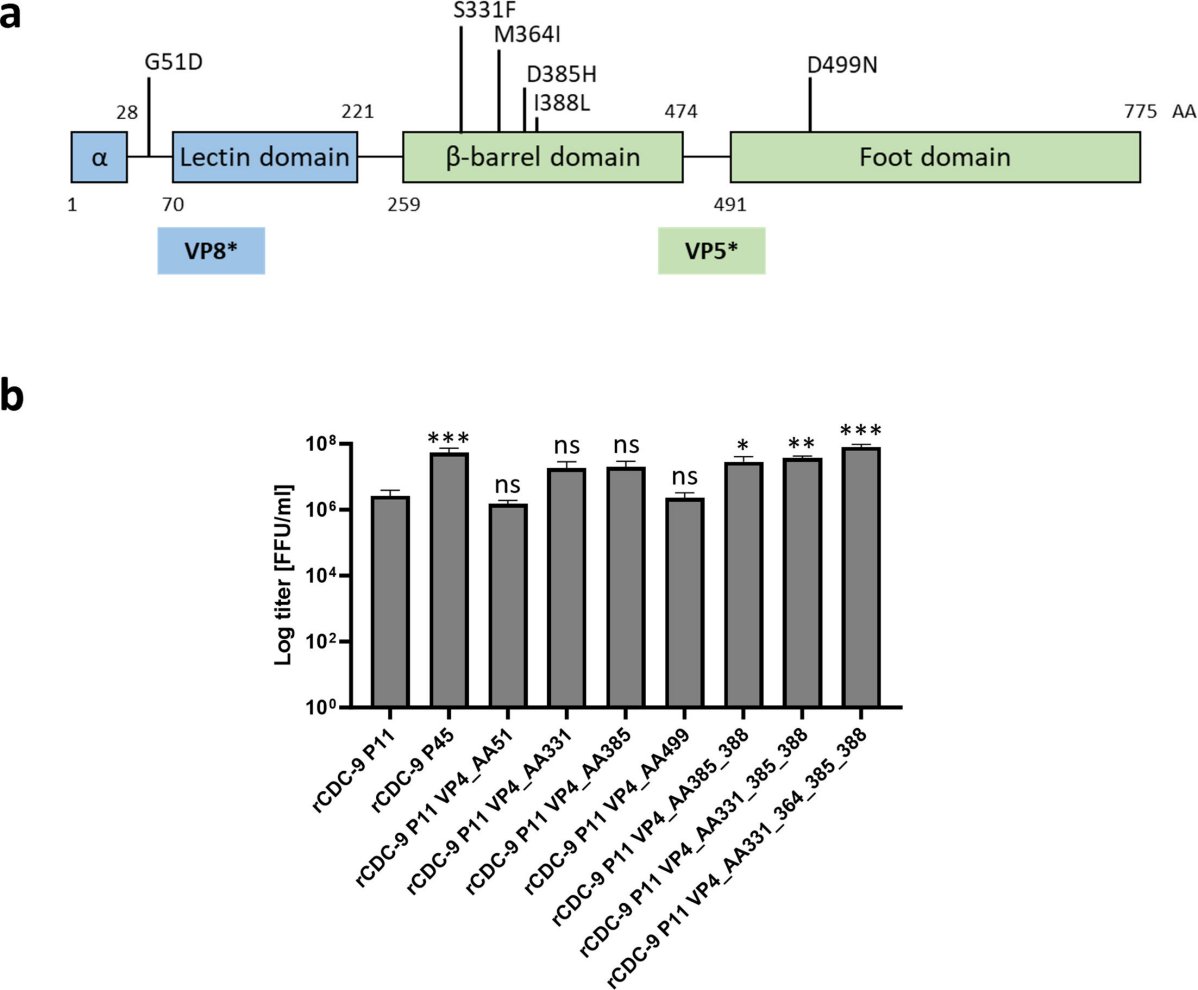
Growth characteristics of rescued CDC-9 strains with VP4 mutations. (**a**) Schematic overview of rotavirus VP4 gene. Amino acid (AA) mutations from stool/P11 in MA104 cells to P45 in Vero cells are indicated on the top. (**b**) Recombinant CDC-9 stains were generated by transfecting BHK-T7 cells and propagated for up to 4 passages in MA104 cells. All recombinant strains were titered and sequenced to confirm the accuracy of point mutation(s). *N* = 3. Shown are mean and SEM. Statistical analysis was performed by One-way ANOVA compared to rCDC-9 P11. Ns, not significant *P* > 0.5. **P ≤ 0.1. ***P ≤ 0.001.

By creating recombinant viruses harboring CDC-9 P45 VP4 on a CDC-9 P11 background, we demonstrated the importance of mutations in VP4 for attenuation *in vivo* and adaptation *in vitro* (Fig. 1). During serial passaging in Vero cells, we observed a total of six functional AA mutations within VP4 (Table 1). To identify which mutation in the VP4 gene of the rotavirus vaccine candidate strain CDC-9 was responsible for growth adaptation *in vitro* and attenuation *in vivo*, we have generated multiple recombinant rotavirus strains with single or multiple point mutations in the VP4 gene. The structural view of VP4 indicated that 5 of the 6 present mutations are within the VP5* region of VP4, whereas only one mutation is in the VP8* region of VP4 (Fig. 2a). We successfully generated constructs of CDC-9 P11 (rCDC-9 P11) and CDC-9 P45 (rCDC-9 P45), single point mutations in VP4 on a P11 backbone (rCDC-9 P11 VP4_AA51, rCDC-9 P11 VP4_AA331, rCDC-9 P11 VP4_AA385, and rCDC-9 P11 VP4_AA499) as well as multiple point mutations in VP4 on a P11 background (rCDC-9 P11 VP4_385_388, rCDC-9 P11 VP4_AA331_385_388, and rCDC-9 P11 VP4_AA331_364_385_388) by reverse genetics and determined their titers at passage 4 in MA104 cells (Fig. 2b). We observed a significant titer difference of rCDC-9 P45, rCDC-9 P11 VP4_AA385_388, rCDC-9 P11 VP4_AA331_385_388, and rCDC-9 P11 VP4_AA331_364_385_388 when compared to rCDC-9 P11. Of note, rCDC-9 P11 VP4_AA51 and rCDC-9 P11 VP4_AA499 showed lower titers than rCDC-9 P45, but comparable titer to rCDC-9 P11. We were not able to generate single point mutants for AA364 and AA388. Sequences and mutations for all recombinant CDC-9 strains generated were confirmed by Next Generation Sequencing (data not shown).

**TABLE 1:** Sequence of mutations occurred during serial passaging of CDC-9 in cell culture^*a*^

Gene	Nt AA	Stool	Cell-passaged virus							
			MA104	Vero						
			P11*	P12	P16	P25	P26	P28	P43	P45
VP4	161 51	G Gly	−	A Asp	A Asp	A Asp	A Asp	A Asp	A Asp	A Asp
	1001 331	C Ser	−	T Phe	T Phe	T Phe	T Phe	T Phe	T Phe	T Phe
	1101 364	G Met	−	−	A Ile	A Ile	A Ile	A Ile	A Ile	A Ile
	1162 385	G Asp	−	C His	C His	C His	C His	C His	C His	C His
	1171 388	A Ile	-	-	-	-	C Leu	C Leu	C Leu	C Leu
	1504 499	G Asp	−	−	−	−	−	−	A Asn	A Asn
	1785 Silent	T −	−	−	−	−	−	−	C −	C −
	2025 Silent	T −	−	−	−	C −	C −	C −	C −	C −
Infectivity (titer FFU/mL)		ND	2.6 × 10^5^	2.5 × 10^4^	ND	1.2 × 10^7^	1.3 × 10^7^	2.7 × 10^7^	4.5 × 10^7^	2.1 × 10^7^
Virulence (shedding)		ND	+++	ND	ND	ND	ND	ND	ND	+
Diarrhea		ND	+++	ND	ND	ND	ND	ND	ND	−

^*a*^ VP4 mutations occurring during serial CDC-9 passages (P) are shown. Nt, nucleotide; AA, amino acid; Gly, glycine; Ser, serine; Met, methionine; Asp, aspartate; Ile, isoleucine; Phe, phenylalanine; His, histidine; Leu, leucine; Asn, asparagine. CDC-9 P11 was generated as a sister strain of CDC-9 P12 in MA104 cells and has been indicated with “*” to demonstrate a sister lineage. ND, not determined. For virulence, the following criteria were established. +++, OD [450 nm] >0.5; ++, OD [450 nm] >0.3 − ≤ 0.5; +: OD [450 nm] ≤0.3. For diarrhea, the following criteria were established. +++, diarrhea score >2 for more than 2 days; −, no diarrhea (score of ≤1 throughout the study).

### Comparison of recombinant CDC-9 strains *in vitro*

**Fig 3:**
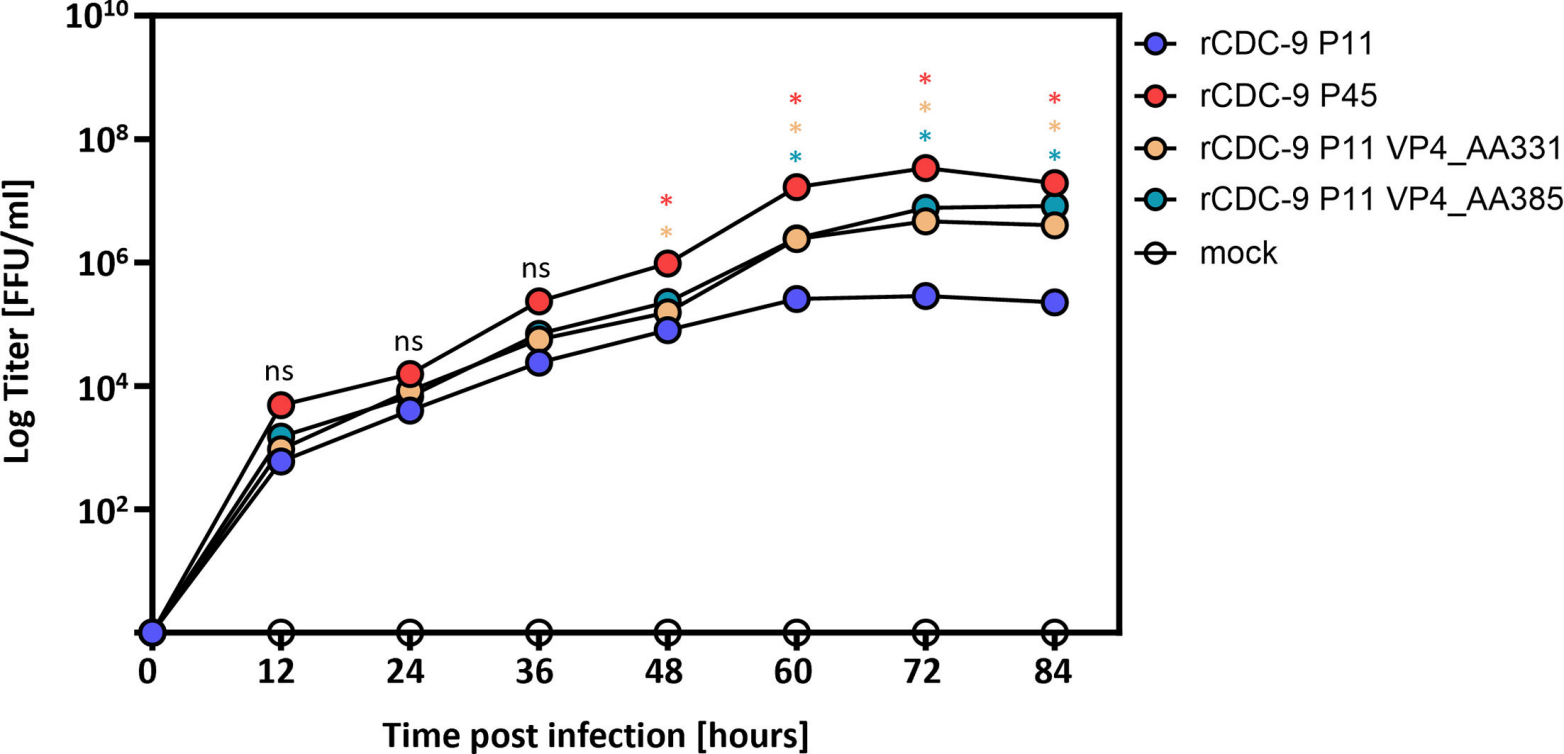
Growth curve of recombinant CDC-9 rotaviruses in human Caco-2 intestinal cells. Human intestinal cells (Caco-2) were infected with an MOI of 0.1 of recombinant viruses for 1 h. At the indicated timepoints, samples were collected, and titration was performed. Representative data (mean and SEM from *N* = 3) are shown from two independent experiments. Infectious titers of rCDC-9 P45 and rCDC-9 P11 VP4 mutants were compared with those of rCDC-9 P11 by two-way ANOVA. Statistics are corresponding to colors in the graph. ns, not significant (*P* ≥ 0.05); *P < 0.05.

In a previous study (22), we showed that native CDC-9 P45 grew to higher titers than CDC-9 P11 in a human intestinal cell line (Caco-2). Here, we used the recombinant CDC-9 strains and VP4 mutant strains to determine if there were differences in growth for these recombinant strains as well. As seen for the original CDC-9 P11 and CDC-9 P45 strains, we showed that recombinant CDC-9 P11 (rCDC-9 P11) grew to significantly lower titers compared to rCDC-9 P45 over the time course of 84 h in Caco-2 cells (Fig. 3). Recombinant viruses with single point mutations at position AA331 or AA385 were able to replicate to titers seen for rCDC-9 P45 indicating that these two mutations are mainly responsible for the adaptation of CDC-9 strain *in vitro*. Recombinant viruses with single point mutations at position 51 or 499 showed comparable growth to rCDC-9 P11 in Caco-2 cells, indicating that these two mutations do not play a role in cell culture adaptation (Fig. S1). Of note, infection with recombinant virus harboring both mutations at position 385 and 388 (rCDC-9 P11 VP4_AA385_388) did not appear to significantly increase the titer compared to rCDC-9 P11 VP4_AA385 alone. Infection with rCDC-9 P11 VP4_AA331_385_388 and rCDC-9 P11 VP4_AA331_364_385_388 showed a slight additive effect on replication. In addition to infectious titers, we examined the sizes of plaques from infections by the original CDC-9 P11 and its VP4 mutants. We observed clear large plaque sizes after infection with native CDC-9 P45 and its mutants rCDC-9 P11 VP4_AA331 and rCDC-9 P11 VP4_AA385 in MA104 cells. By contrast, we did not observe any plaques by CDC-9 P11 (data not shown). These data indicate that mutations at position AA331 and AA385 are critical for optimal adaptation and growth *in vitro*.

### Identification of molecular signatures involved in CDC-9 attenuation and pathogenesis in neonatal rats

**Fig 4:**
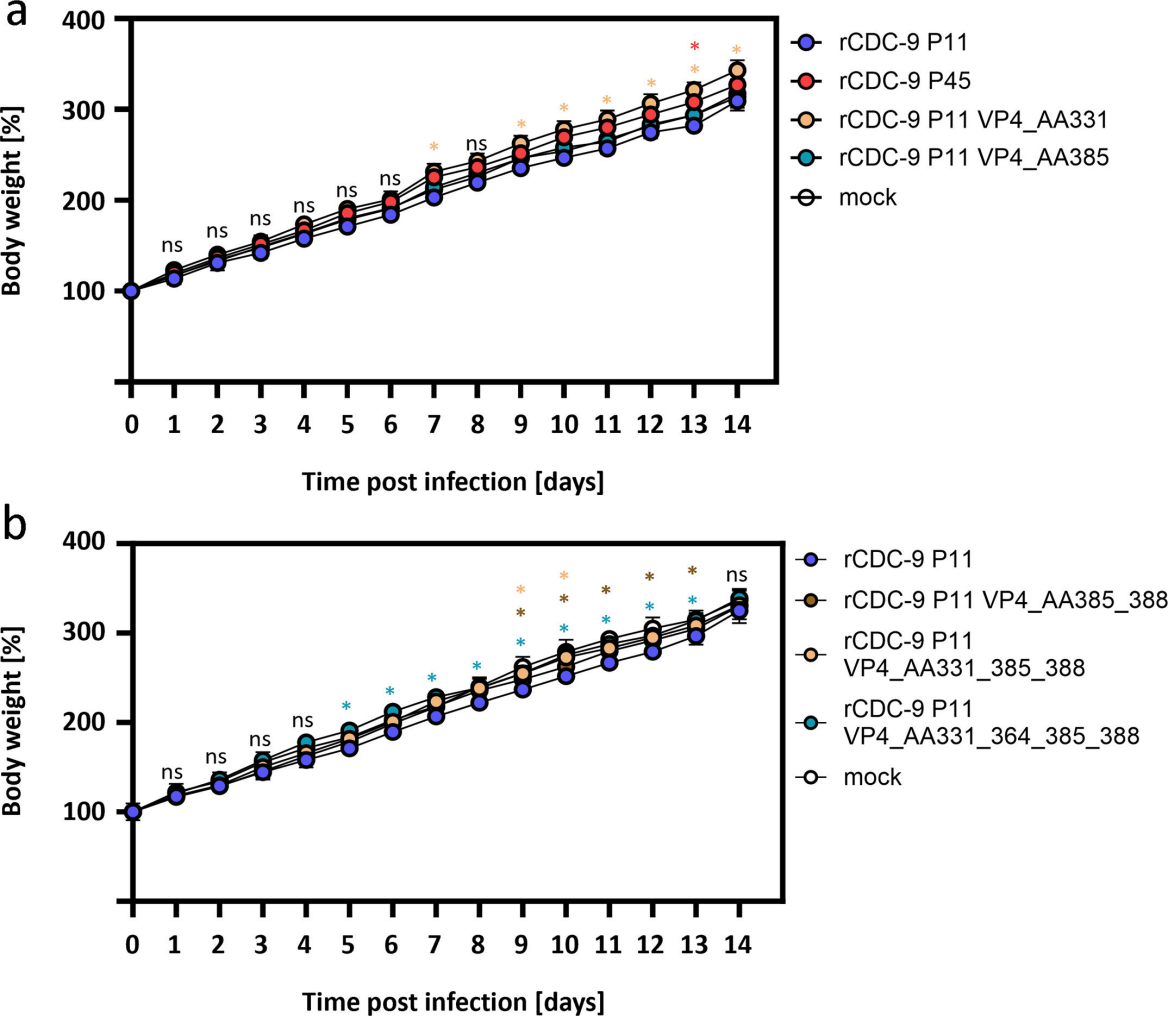
Body weight comparison of neonatal rats after infection with single or multiple mutations in VP4 of recombinant CDC-9 rotaviruses. (**a**) Five-day-old neonatal rats were infected with 1 × 10^7^ FFU of recombinant CDC-9 strains rCDC-9 P11, rCDC-9 P45, rCDC-9 P11 VP4_AA331, and rCDC-9 P11 VP4_AA385 or mock infection. Shown are means and SEM. *N* = 8. (**b**) Five-day-old neonatal rats were infected with 1 × 10^7^ FFU of recombinant strains rCDC-9 P11 (*N* = 4), rCDC-9 P11 VP4_AA385_388 (*N* = 8), rCDC-9 P11 VP4_AA331_385_388 (*N* = 8), and rCDC-9 P11 VP4_AA331_364_385_388 (*N* = 8) or mock infected (*N* = 5). Shown are means and SEM. Analysis was done by two-way ANOVA test to compare body weight gains between mutant groups and the rCDC-9 P11 group. Statistics correspond to colors in the graphs. Ns, not significant (*P* ≥ 0.05); *P < 0.05.

Since we previously observed no effect of CDC-9 P45 VP4 on bodyweight gain but significantly reduced virus shedding, we next assessed which of these VP4 mutations were important for attenuation of the strain CDC-9 *in vivo* by using a neonatal rat model. Infection with rCDC-9 P11 led to significantly less gain in body weight from day 6 to 13 compared to infection with rCDC-9 P45 and mock-inoculated animals (Fig. 4a). Infection with rCDC_9 P11 VP4_AA331 and rCDC-9 P11 VP4_AA385 showed normal gain in body weight comparable to rCDC-9 P45 and mock control. Combination mutants rCDC-9 P11 VP4_AA331_385_388 and rCDC-9 P11 VP4_AA331_364_385_388 also showed significantly greater gain in body weight than rCDC-9 P11 (Fig. 4b).

**Fig 5:**
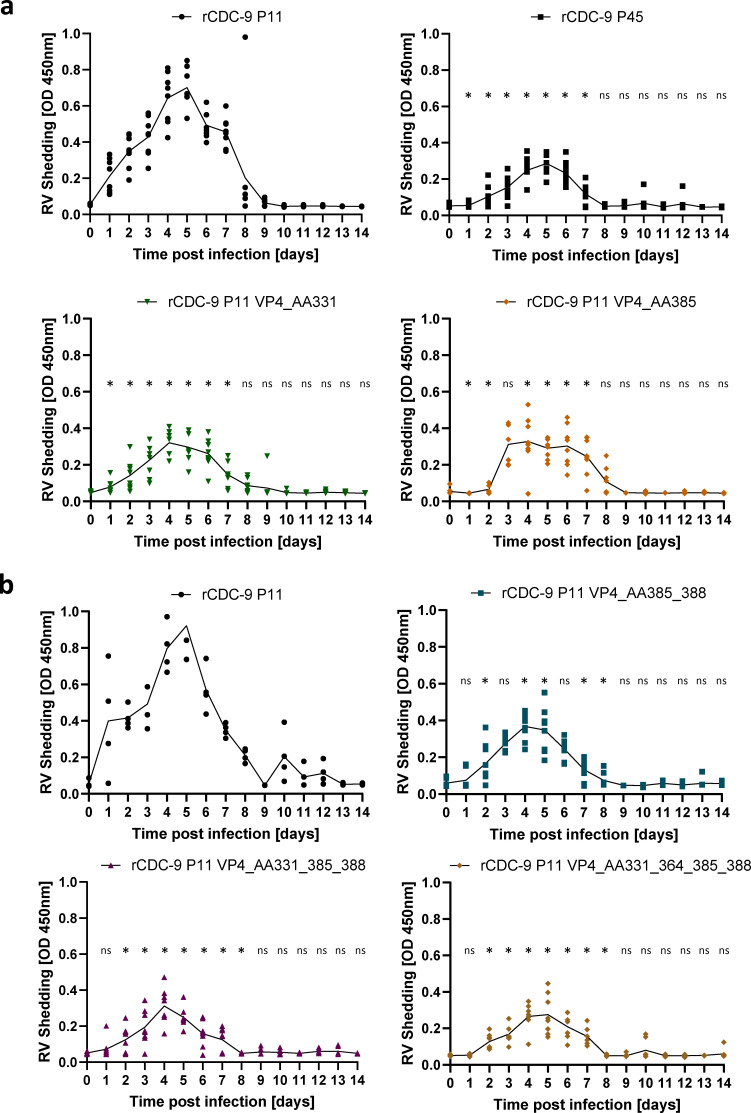
Reduced RV shedding after infection with recombinant CDC-9 mutants harboring VP4 mutations at positions AA331 and AA385. Five-day-old neonatal rats were infected with 1 × 10^7^ FFU of the indicated recombinant CDC-9 strains (**a**) rCDC-9 P11, rCDC-9 P45, or single mutations in VP4 gene or (**b**) rCDC-9 P11 and mutants harboring 2, 3, or 4 mutations in VP4. For (A), *N* = 8. For (B), rCDC-9 P11 *N* = 4; rCDC-9 P11 VP4_AA385_388, rCDC-9 P11 VP4_AA331_385_388, and rCDC-9 P11 VP4_AA331_364_385_388 *N* = 8; mock *N* = 5. Shown are mean and SEM. Analysis was done by two-way ANOVA test to compare shedding between mutant groups and the rCDC-9 P11 group. *, *P* < 0.05. ns, not significant (*P* ≥ 0.05).

To identify the effect of VP4 and its mutants on disease progression and rotavirus shedding, we measured rotavirus shedding over 14 days in neonatal rats. rCDC-9 P11 induced a high level of shedding with a peak shedding between day 4 and 5 (Fig. 5a). rCDC-9 P11 further induced diarrhea in 4 out of 10 animals 2–3 days post inoculation, no diarrhea was observed in neonatal rats infected with other recombinant viruses (Table 2). Infection with rCDC-9 P45 induced significantly less shedding compared to rCDC-9 P11 with peak shedding from day 4 to 6. A similar pattern was seen in animals infected with rCDC-9 P11 VP4_AA331 or rCDC-9 P11 VP4_AA385 with significantly reduced shedding compared to rCDC-9 P11. However, the peak shedding in animals infected with rCDC-9 P11 VP4_AA385 lasted from day 3 to day 7, whereas peak shedding after infection with rCDC_9 P11 VP4_AA331 had a shorter peak between day 4 and day 6. Similar to the *in vitro* data, mutations at position AA51 and AA499 did not appear to significantly change the virulence of the viruses compared to rCDC-9 P11 (Fig. S2). We observed elevated levels of shedding in animals infected with rCDC-9 P11 VP4_AA51 or rCDC-9 P11 VP4_AA499 and rCDC-9 P11 with a peak on days 4 and 5.

**TABLE 2:** Summary of *in vitro* and *in vivo* results of different recombinant CDC-9 strains^*a*^

		rCDC-9						
		P11	P45	P11 VP4_P45	P11 VP4_AA331	P11 VP4_AA385	P11 VP4_AA331_ 385_388	P11 VP4_AA331_364_ 385_388
*In vitro*	Infectivity (titer FFU/mL)	2.67 × 10^6^	5.33 × 10^7^	3.65 × 10^7^	1.85 × 10^7^	1.97 × 10^7^	3.72 × 10^7^	8.0 × 10^7^
*In vivo*	Virulence (shedding)	+++	+	+	+	+	+	+
	Diarrhea	++	−	−	−	−	−	−
	Cytokine profile	Pro	Anti	ND	Anti	Pro	Anti	Anti

^*a*^ *In vitro* infectivity (titer in MA104 cells) and *in vivo* response (viral shedding, diarrhea, and cytokine profile in neonatal rats) are shown for recombinant CDC-9 variants. ND, not determined. For virulence, the following criteria were established. +++, OD [450 nm]; >0.3 − ≤ 0.5; +, for diarrhea, the following criteria were established. +++, diarrhea score >2 for more than 2 days; −, no diarrhea (score of ≤1 throughout the study). For cytokines, “pro” refers to predominantly pro-inflammatory cytokine response and “anti” refers to predominantly anti-inflammatory cytokine response.

To examine whether multiple mutations in VP4 had an additive effect, rats were infected with recombinant CDC-9 with two, three, or four mutations in VP4 (Fig. 5b). rCDC-9 P11 double VP4 mutants AA385 and AA388 induced level of shedding comparable to the single mutant AA385. rCDC-9 P11 triple VP4 mutants AA331, AA385, and AA388 and quadruple mutants AA331, AA364, AA385, and AA388 induced comparable levels of shedding with peak at day 4 or 5, which appeared to be slightly lower than that from the double mutant. These results showed that double mutations at VP4 AA331 and AA385 appeared to have an additive effect on the level of attenuation of this G1P[8] rotavirus, whereas mutation at VP4 AA388 seemed to have a small added effect on its further attenuation. Additional mutation at position AA364 (rCDC-9 P11 VP4_AA331_364_385_388) did not appear to significantly reduce the shedding further.

### Cytokine response after infection with recombinant CDC-9 strains and mutants

**Fig 6:**
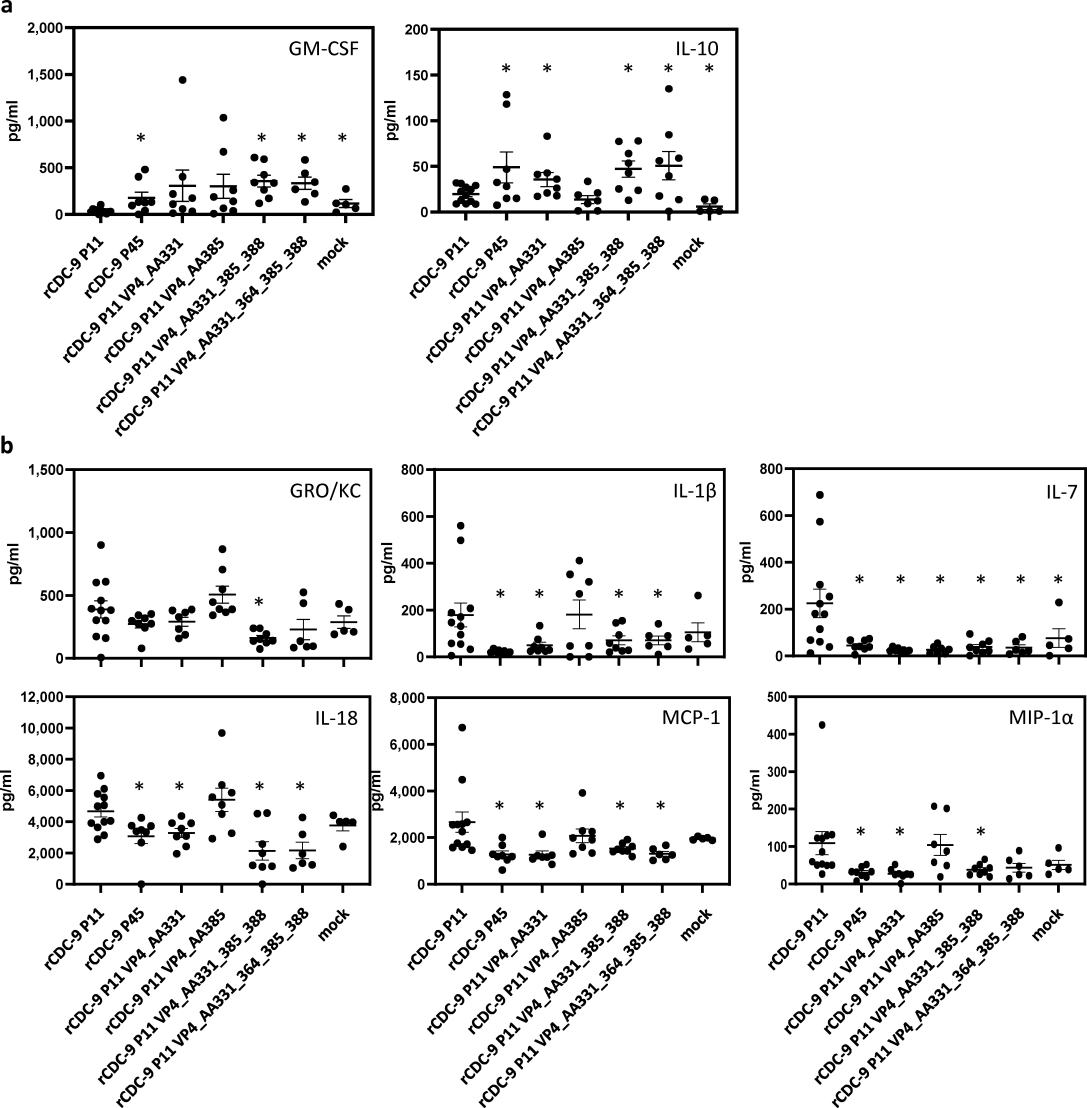
Cytokine profile after infection with recombinant CDC-9 underlines the importance of VP4 AA331 change in induction of anti-inflammatory cytokines for attenuation. Five-day-old neonatal rats were infected with 1 × 10^7^ FFU of the indicated viruses. Serum samples were collected on day 21 post infection. Cytokines were measured by using the BioPlex Rat Cytokine 23-plex assay. (**a**) Anti-inflammatory cytokines GM-CSF, IL-4, and IL-10 are shown. (**b**) Pro-inflammatory cytokines GRO/KC, IL-1β, IL-7, IL-18, and chemokines MCP-1 and MIP-1α are shown. Unpaired *t*-test was used to compare levels of cytokines between mutant groups and the rCDC-9 P11 group. *P < 0.05; If nothing indicated, not significant (*P* ≥ 0.05). *N* = 12 (rCDC-9 P11); *N* = 5 (mock); *N* = 8 (remaining mutants) except rCDC-9 P11 VP4_AA331_364_385_388 (*N* = 6).

Cytokines and chemokines play a pivotal role in disease progression and rotavirus shedding (26). We analyzed cytokine and chemokine profiles in neonatal animals after infection with different recombinant rotaviruses (Fig. 6). We showed that anti-inflammatory cytokine Interleukin (IL)-10 and immune-modulatory granulocyte macrophage colony-stimulating factor (GM-CSF) were significantly upregulated after infection with rCDC-9 P45 compared to rCDC-9 P11 (Fig. 6a). Additionally, we observed significant upregulation of GM-CSF after infection with rCDC-9 P11 VP4_AA331_385_388 and rCDC-9 P11 VP4_AA331_364_385_388. We also observed a significant increase of IL-10 in rCDC-9 P11 VP4_AA331-infected animals compared to rCDC-9 P11 VP4_AA385 infected rats. On the other hand, pro-inflammatory cytokines IL-1β, IL-7, IL-18, and chemokines MCP-1 and MIP-1α were upregulated after infection with rCDC-9 P11 compared to rCDC-9 P45 (Fig. 6b). We showed that infection with rCDC-9 P45 led to reduced expression of pro-inflammatory cytokines IL-2 and significantly reduced expression of IL-12 compared to infection with rCDC-9 P11 (Fig. S3). We showed that rCDC-9 P11 VP4_AA385 induced comparable levels of IL-1β, IL-18, MCP-1, and MIP-1α to as rCDC-9 P11 indicating that this mutation was not involved in modulating these cytokines *in vivo*. Of note, no significant difference was found for type II interferon expression in any of the VP4 mutants tested (data not shown).

## DISCUSSION

In the present study, we have comprehensively analyzed the mutations within rotavirus VP4 gene that occurred during serial passaging of the human vaccine strain CDC-9 in Vero cells and identified for the first time, two molecular signatures in the membrane fusion domain of VP5 that facilitated adaptation *in vitro* and attenuation *in vivo*. It has been previously shown that VP4 is the key factor facilitating replication in cell culture when examining heterologous recombinant rotavirus strains with human or murine VP4 on a simian rotavirus backbone (25, 27, 28). We also showed that infection of neonatal rats with a simian VP4 (RRV) on a human backbone (rCDC-9 P11) leads to significantly higher shedding in neonatal rats compared to the parental human rCDC-9 P11 rotavirus infection (Fig. 1b). In addition, when using rCDC-9 P11 (wild-type) backbone with VP4 from CDC-9 P45 (attenuated), we were able to recreate findings we observed with a full recombinant and cell culture-derived attenuated CDC-9 P45. We further showed that rCDC-9 P11 VP4_P45 and rCDC-9 P45 grew to significantly higher titers in simian (MA104) and human (Caco-2) cell lines compared to rCDC-9 P11 (Fig. 2b and 3). These data support a previously published study from our lab that original, cell culture-adapted CDC-9 P45 grew to significantly higher titers than CDC-9 P11 (22). Our findings demonstrate the primary importance of VP4 in adaptation to high growth in cell culture despite a few mutations occurring in other rotavirus genes. One early study examined the effect of mutations within the NSP4 gene on the attenuation of human rotavirus vaccine Rotarix, but no correlation was identified (29).

Previous studies analyzed the effect of whole VP4 gene substitution and did not identify specific mutations within VP4 by reverse genetics (25, 28). When analyzing mutations of VP4 in circulating or passaged strains, most mutations appeared within the VP5* region, especially between AA360 and AA400 (30–32). Region AA382-400 of VP4 has been defined as the membrane interaction loop in VP5 (33) with the fusion domain at AA385. Substitutions in this region may favor a growth advantage in cell culture and render the virus attenuated in humans. Five mutations appear in the vaccine strain Rotarix within VP4, namely, G51D, L167F, S331F, D385Y, and N695I (13). Mutations at positions 51, 331, and 385 are found conserved in Rotarix and CDC-9 after serial passaging in cell culture (21, 22) (Table 1). Rotavirus entry is highly dependent on the fusion domain in VP5* (AA384–AA404) and AA changes within this region can result in conformational changes in VP4 (34). The position AA385 was identified by several other studies as a mutation following serial passaging (30, 32, 35, 36) of human rotavirus, while high conservation of 385D was reported in wild-type P[8] rotavirus and not in cell culture passaged strains (37). However, these findings only identify associations between AA mutation at this one position and strain adaptation and attenuation.

Nevertheless, these published data with a single point mutation at AA385 were in agreement with increased replication capacity in cell culture and reduced infectivity in neonatal rats in the present study. We further observed an additive effect of the double mutations at AA331 and AA385 on attenuation, which agreed with the data that these two mutations appeared together at passage 13 (7 passages in MA104 cells and 6 in Vero cells) when the strain was adapted to grow in Vero cells. In addition, we observed mutations at positions AA388 and AA499 that appeared at passage 26 and 43 (19 and 36 in Vero cells), respectively (Table 1). The appearance of the mutation in Vero cell-adapted virus can be directly linked to an increased titer (~10^7^ FFU/mL at P25) compared to MA104-passaged CDC-9 P11 (titer ~10^5^ FFU/mL; Table 1). Infectious titer did not increase further following the appearance of these late mutations since the titer ranged from 1.3 × 10^7^ FFU/mL to 4.5 × 10^7^ FFU/mL between passages 26 and 45. We demonstrated a negative association between cell culture passages and virus virulence; CDC-9 P11 induced high levels of virus shedding and diarrhea in animals, while CDC-9 P45-infected animals had significantly reduced viral shedding and no diarrhea (Table 1), confirming findings in a previous study (22).

We were not able to generate recombinant CDC-9 strains with single point mutations at position AA364 and AA388. Since those mutations occurred after the initial AA change at positions 51, 331, and 385 at passage 13, we speculate that there could be a structural change within the VP4 protein that induced additional mutations at AA364 and AA388 to produce a stable VP4 protein. For CDC-9 P45, we observed that the VP4 protein appeared in an upright conformation compared to VP4 from CDC-9 P11 (38). The appearance of the upright confirmation of VP4 was explained by the mutations mainly at AA331, AA385, and AA388 stabilizing the apex of VP4 spikes. These data were in accordance with a recently published study that compared AA mutations in various rotavirus genotypes and showed that mutations accumulate between AA380 and AA390 (31). No mutations in AA331 region were seen in that study for the analyzed rotavirus genotypes G1P[8], G3P[8], G9P[8], G12P[8], and G2P[4]. However, that study was done by passaging rotavirus genotypes in primary RhMK cells (up to 5 passages) followed by passaging in MA104 cells (up to 10 passages), whereas we saw mutations accumulating after adapting and passaging in Vero cells, a cell substrate commonly used for vaccine manufacturing. We showed no additive effect of mutations AA385 and AA388 (rCDC-9 P11 VP4_AA385_388); however, once we introduced AA331 (rCDC-9 P11 VP4_AA331_385_388) and AA364 (rCDC-9 P11 VP4_AA331_364_385_388), we were able to see comparable titers *in vitro* (Caco-2 cells, Fig. S1) as well as normal body weight gain and reduced shedding in neonatal rats (Fig. 4b and 5b).

Cytokines are an important tool to target and help clear viral infections. Various cytokines have been reported after infection with different rotaviruses. Pro-inflammatory cytokines and chemokines like IL-1β, IL-8, GRO-α, RANTES, MIP-1α, MCP-1, and IFN-γ have been detected in cell culture supernatants and plasma and mucosal surfaces after infection in humans (39–42). We detected induction of pro-inflammatory cytokines and chemokines (e.g., IL-1β and MIP-1α; Fig. 6b) in neonatal rats after infection with rCDC-9 P11 and a shift to anti-inflammatory cytokines (e.g., IL-10, Fig. 6a) after infection with attenuated rCDC-9 P45 or VP4 mutants harboring AA331 mutations, either single or in combination (VP4_AA331, VP4_AA331_385_388, and VP4_AA331_364_385_388). These data highlight the association of mutations at AA331 and reduced viral shedding as well as down regulated inflammatory response. Of note, infection with rCDC-9 P11 VP4_AA385 did not downregulate the pro-inflammatory response for selected cytokines (e.g., IL-1β, IL-18 MIP-1α) indicating that AA385 might be involved in reduced viral shedding *in vivo* but did not modulate inflammatory responses as seen with rCDC-9 P11 VP4_AA331. Further studies will need to investigate the effects of AA385 and other mutations on host responses *in vivo* to fully understand the immunological properties of attenuated rotavirus vaccines. The present study examined cytokine profiles in sera of animals 21 days post infection. Due to the limited number of neonatal rats available, no cytokine analysis was conducted for early time points, which should be done in future studies.

For CDC-9, we have observed major changes in amino acid properties for position 331 (S331F, hydrophilic to hydrophobic) and a change from hydrophilic acidic to hydrophilic basic amino acid at position 385 (D385H) (Table 1), resulting in increased growth in cell culture (MA104 and Caco-2) and reduced pathogenicity in neonatal rats (Table 2). In a previously conducted study analyzing the protein structure of VP4 in CDC-9 P11 and CDC-9 P45, we demonstrated that the VP5* domain of CDC-9 P45 remained stable in an upright position, indicating a fully infectious virion, compared to CDC-9 P11 which was unstable and unable to mediate cell entry (38). These data were consistent with the lower infectivity *in vitro* of CDC-9 P11 as well as rCDC-9 P11. During this study, we found that a mutation in VP4 at position AA51, AA331, or AA385 was likely to stabilize the tip of the VP4 spike and, therefore, result in the formation of triple-layered CDC-9 P45 particles. In accordance with these previous findings, our results from rCDC-9 P11 strains with single point mutations at position AA331 or AA385 showed a significant increase in viral growth *in vitro* (Fig. 3) and reduced viral shedding *in vivo* (Fig. 5) compared to rCDC-9 P11. In contrast, rCDC-9 P11 VP4_AA51 did not result in an increased viral growth *in vitro* (Fig. S1) or decreased viral shedding *in vivo* (Fig. S2) compared to rCDC-9 P11. Consequently, we concluded that mutation at either AA331 or AA385, or both were responsible for attenuation and adaptation. When analyzing recombinant variants with multiple point mutations (Fig. S1 for *in vitro* results and Fig. 5 for *in vivo* results), we demonstrated that the combination of mutations at position AA331, AA385, and AA388 appeared to result in comparable results to the attenuated rCDC-9 P45. Since mutations at position AA331 and AA385 appeared at the same time during serial passaging and the two mutants singularly or in combination induced *in vitro* and *in vivo* responses comparable to rCDC-P45 remained to be determined whether all three mutations at AA331, AA385, and AA388 are needed to work together to fully attenuate CDC-9. We showed that rCDC-9 P11 VP4 AA331 and rCDC-9 P11 VP4 AA385 mutants induced predominantly anti-inflammatory or suppressed pro-inflammatory response compared with a general pro-inflammatory state in rCDC-9 P11 infected animals. This shift from pro-inflammatory response against wild-type CDC-9 P11 to anti-inflammatory state or reduced pro-inflammatory response against late passaged CDC-9 P45 or VP4 mutants might explain dramatic reduction in shedding and their apparent attenuation in neonatal rats. Future studies will be conducted to determine the three-dimensional structures of VP4 mutants containing the single point mutations (rCDC-9 P11 VP4_AA331, rCDC-9 P11 VP4_AA385, or rCDC-9 P11 VP4_AA388) and multiple point mutations by cryo-electron microscopy and examine virus-host interactions to elucidate mechanisms of mutations and associated functional changes.

In summary, we were successful in generating recombinant human rotaviruses based on the sequence of the vaccine candidate CDC-9 wildtype (P11) and attenuated (P45) strain. We demonstrated that mutations at positions AA331 and AA385 within the VP5* region of the VP4 gene of this vaccine strain were crucial in mediating adaption in cell culture and attenuation as well as downregulated inflammatory response in neonatal rats. These findings together with conserved mutations at AA331 and AA385 from the licensed Rotarix vaccine allowed us to identify the molecular signatures that define the attenuation and safety of human rotavirus vaccine G1P[8] strains. Studies are in progress to examine specific mutations in VP4 and other genes of non G1P[8] strains (e.g., DS-1 like G9P[6]) and their effect on virus attenuation, pathogenesis, and safety in animals and children.

## MATERIALS AND METHODS

### Cells and viruses

African green monkey kidney epithelial cell line MA104 (ATCC CRL-2378.1) was passaged in 199 medium (Sigma Aldrich, St. Louis, MO, USA) supplemented with 10% fetal bovine serum (FBS, one shot, Gibco), 100 IU penicillin/mL, 100 µg/mL streptomycin, and 0.292 mg/mL L-glutamine as previously described (25). A baby hamster kidney fibroblast (BHK) cell line stably expressing T7 RNA polymerase BHK-T7 was kindly gifted by Dr. Buchholz at the NIH and previously described (43). BHK-T7 cell line was cultivated in Dulbecco’s modified Eagle’s medium (DMEM) supplemented with 10% FBS, 100 IU penicillin/mL, 100 µg/mL streptomycin, 0.292 mg/mL L-glutamine, and 0.2 µg/mL G-418 (Promega, Madison, WI, USA). A human intestinal carcinoma cell line Caco-2 (ATCC HTB-37) was passaged in Eagle’s minimum essential medium (EMEM) supplemented with 20% FBS.

Infection of Caco-2 cells for growth curve analysis was performed as previously described (22). In brief, Caco-2 cells were seeded in 24-well plates for 5 days and infected with the indicated viruses at MOI 0.1 for 1 h. After removal of inoculum, fresh serum-free EMEM containing 10 µg/mL porcine trypsin (Gibco) was added and samples collected at the indicated time points and used for virus titration.

Original CDC-9 virus was isolated in 2003 from a 5-month-old boy hospitalized in the United States that presented with acute diarrhea (21). CDC-9 was passaged for up to 7 passages on MA104 cells followed by passaging on Vero cells for up to 45 passages (P45) for vaccine seed virus preparation. To increase titer of early passage virus, the common progenitor of the vaccine seed virus CDC-9 P4 was passaged in MA104 cells up to P11/12 separately. For large-scale virus production, MA104 (for CDC-9 P11/12) or Vero (for CDC-9 P45) cells were seeded in roller bottles until confluency is reached and infected at MOI 0.1 for 2–3 h with the respective viruses. After the addition of fresh serum-free DMEM containing 30 µg/mL porcine trypsin, roller bottles were incubated for 3–5 days until cytopathic effect (CPE) was observed. Roller bottles were placed at −80°C, freeze-thawed three times and clarified by centrifugation at 8,000 rpm for 30 min. Clarified CDC-9 strains were aliquoted and stored at −65 to −95°C for subsequent use. Uninfected MA104 cells in roller bottles were processed in the same manner for use as mock infection.

### Virus titration

Titration of original and recombinant viruses was performed by using an Immunospot assay as previously described (22). In brief, MA104 cells were seeded in 96-well plates and cultivated until confluency was reached. Cells were washed with serum-free Iscove’s modified Dulbecco medium (IMDM, Gibco) and serial dilution of viruses was performed in serum-free IMDM also. Cells were fixed 18 h after infection, and plates were incubated with a rabbit anti-Wa antibody, followed by incubation with a peroxidase-labeled goat-anti rabbit IgG antibody (KPL) and staining with True Blue Peroxidase Substrate (VWR, Radnor, PA, USA). Plates were scanned by using a CTL analyzer (Cellular Technology, Kennesaw, GA, USA). Stained cells over two dilutions were counted and the average number of viruses per milliliter was calculated.

### Plasmids

Human CDC-9 plasmid collection (pT7-CDC-9 P11 VP1, pT7-CDC-9 P11 VP2, pT7-CDC-9 P11 VP3, pT7-CDC-9 P11 VP4, pT7-CDC-9 P11 VP6, pT7-CDC-9 P11 VP7, pT7-CDC-9 P11 NSP1, pT7-CDC-9 P11 NSP2, pT7-CDC-9 P11 NSP3, pT7-CDC-9 P11 NSP4, pT7-CDC-9 P11 NSP5, pT7 CDC-9 P45 VP2, pT7 CDC-9 P45 VP4, pT7 CDC-9 P45 VP7, pT7 CDC-9 P45 NSP1, pT7-CDC-9 P11 VP4_AA51, pT7-CDC-9 P11 VP4_AA331, pT7-CDC-9 P11 VP4_AA385, pT7-CDC-9 P11 VP4_AA499, pT7-CDC-9 P11 VP4_AA385_388, pT7-CDC-9 P11 VP4_AA331_385_388, and pT7-CDC-9 P11 VP4_AA331_364_385_388) was commercially synthesized (GenScript, Piscataway, NJ). Simian SA11 plasmid collection (pT7-SA11_VP1, pT7-SA11_VP2, pT7-SA11_VP3, pT7-SA11_VP4, pT7-SA11_VP6, pT7-SA11_VP7, pT7-SA11_NSP1, pT7-SA11_NSP2, pT7-SA11_NSP3, pT7-SA11_NSP4, and pT7-SA11_NSP5) was originally made by Takeshi Kobayashi (Research Institute for Microbial Diseases, Osaka University, Japan) and obtained from Addgene (44). Plasmid pCMVScript-NP868R-(G4S)4-T7RNAP (C3P3-G1), which autonomously synthesize viral mRNAs containing the main post-transcriptional modifications, was kindly provided by Dr. Jais (Eukarÿs, Evry, France) (45). Plasmid purification was performed using Qiagen EndoFree Plasmid Maxi Kit (Qiagen, Hilden, Germany) according to manufacturer’s instructions. Recombinant mono-reassortment viruses on CDC-9 P11 background with different VP4 genes were received from Stanford University, namely, rCDC-9 P11 VP4_RRV, rCDC-9 P11 VP4_SA11, rCDC-9 P11 VP4_UK, and rCDC-9 P11 VP4_P45 (VP4 gene with all six mutations seen in CDC-9 P45 VP4 was used on a CDC-9 P11 background).

### Reverse genetics

Recombinant rotavirus particles were generated by an improved reverse genetics protocol as previously described (25). In brief, BHK-T7 cells were seeded in 12-well plates and cultivated until 80% confluency was reached. 0.4 µg of 9 rotavirus plasmids (all except NSP2 and NSP5), 1.2 µg of rotavirus plasmids NSP2 and NSP5, and 0.8 µg of helper plasmids C3P3-G1 were mixed in Gibco OPTI-MEM I Reduced Serum Medium (Fisher Scientific, Waltham, MA, USA) and transfected with TransIT-LT1 (Mirus Bio) into BHK-T7 cells. Twenty-four hours after transfection, medium was replaced with serum-free DMEM. Forty-eight hours after transfection, MA104 cells were added to transfected BHK-T7 cells and cultured in the presence of 0.5 µg/mL trypsin. Rescued viruses were amplified by passaging in MA104 cells up to four times. To generate VP4 mutant viruses, we replaced P11 VP4 or P45 VP4 with the appropriate VP4 plasmids containing single or multiple point mutations. Generated recombinant rotavirus sequences were confirmed by next-generation sequencing.

### Next-generation sequencing of virus strains

Viral RNA from rotavirus-infected cell culture was extracted using the Qiagen Viral RNA Kit according to manufacturer’s instructions followed by a DNase I treatment (Qiagen, Hilden, Germany) and cleanup with QIAquick PCR purification kit. Total RNA (250 ng) was used as input for rRNA Depletion Kit (NEB, Ipswich, MA, USA) followed by the NEB Ultra II RNA library preparation kit according to the manufacturer’s instructions. Sequencing was performed using an Illumina MiSeq V2 reagent kit 500 cycles (Illumina, San Diego, CA, USA). Data were analyzed by CLC Genomics Workbench (V21 and V22, Qiagen, Hilden, Germany).

### Infection of neonatal rats

Timed-pregnant Lewis rats that were seronegative for RV were purchased from Charles River (Wilmington, MA, USA). One day after birth, neonatal pups were randomly distributed to a litter size of 4–10 pups per mother depending on experiment. Animal numbers for each experiment are given in the respective figure legends. Five-day-old neonatal rats were infected by oral gavage with 1 × 10^7^ FFU with the indicated viruses diluted in serum-free IMDM or given mock control (clarified supernatant of uninfected MA104 cells). Animals were checked daily for 14 days post infection for body weight and diarrhea score. Diarrhea scoring was as follows. 0: normal feces; 1: unusually loose, yellow stool; 2: mucus with liquid stool, some loose stool; 3: totally loose yellow-green feces; 4: high amount of watery feces. Rectal swabs were collected in 0.5 mL Premier Rotaclone Diluent daily for 14 days post infection and stored at −65 to −95°C before testing. Shedding was analyzed by testing rectal swabs with Premier Rotaclone EIA (Meridian, Cincinnati, OH, USA) according to the manufacturer’s instructions. On day 21 post infection, all animals were euthanized, whole blood collected and allowed to clot at room temperature for 30 min before centrifuging for 10 min at 2,000 × *g* in a refrigerated centrifuge. Serum was aliquoted into clean tubes for subsequent assays and stored at −80°C.

### Cytokine assay

Cytokine and chemokine expression in rat serum was performed using Bioplex Pro Rat Cytokine 23-plex Assay (G-CSF, GM-CSF, GRO/KC, IFN-γ, IL-1α, IL-1β, IL-2, IL-4, IL-5, IL-6, IL-7, IL-10, IL-12 (p70), IL-13, IL-17A, IL-18, M-CSF, MCP-1, MIP-1α, MIP-3α, RANTES, TNF-α, and VEGF) according to manufacturer’s instructions. Samples were analyzed by using BioPlex 200 System (BioRad, Hercules, CA, USA) and Bioplex Manager Software (BioRad, Hercules, CA, USA).

### Statistical analysis

Graphs were generated, and statistical analyses were performed by using GraphPad Prism V10 (GraphPad Software, La Jolla, CA, USA). Two-way analysis of variance (ANOVA) was used to determine statistical significance of rotavirus shedding and body weight gain in neonatal rats. For all other analyses, two-tailed Student’s *t*-test was performed. *P* values < 0.05 were considered statistically significant and are indicated with an asterisk.

## Data Availability

Data generated are available in the article and associated supplemental tables and figures. The plasmid sequences used in this study are available upon request.

## SUPPLEMENTAL MATERIAL

Fig. S1 jvi.01067-25-s0001.tif In vitro growth curve of recombinant CDC-9 viruses in human intestinal cells.

Fig. S2 jvi.01067-25-s0002.tif Elevated RV shedding after infection with recombinant CDC-9 strains harboring VP4 mutations at positions AA51 and AA499.

Fig. S3 jvi.01067-25-s0003.tif Cytokine profile after infection with recombinant CDC-9 underlines the importance of VP4 AA331 change in induction of anti-inflammatory cytokines for attenuation.

ASM does not own the copyrights to Supplemental Material that may be linked to, or accessed through, an article. The authors have granted ASM a non-exclusive, world-wide license to publish the Supplemental Material files. Please contact the corresponding author directly for reuse.
