# Supplementary figures and images for: Mutations of two amino acids in VP5 mediate the attenuation of human rotavirus vaccine: evidence from in vitro and in vivo studies
Source: J Virol. 2025 Oct 8;99(11):e01067-25. doi: 10.1128/jvi.01067-25 (PMC12645992; doi:10.1128/jvi.01067-25)

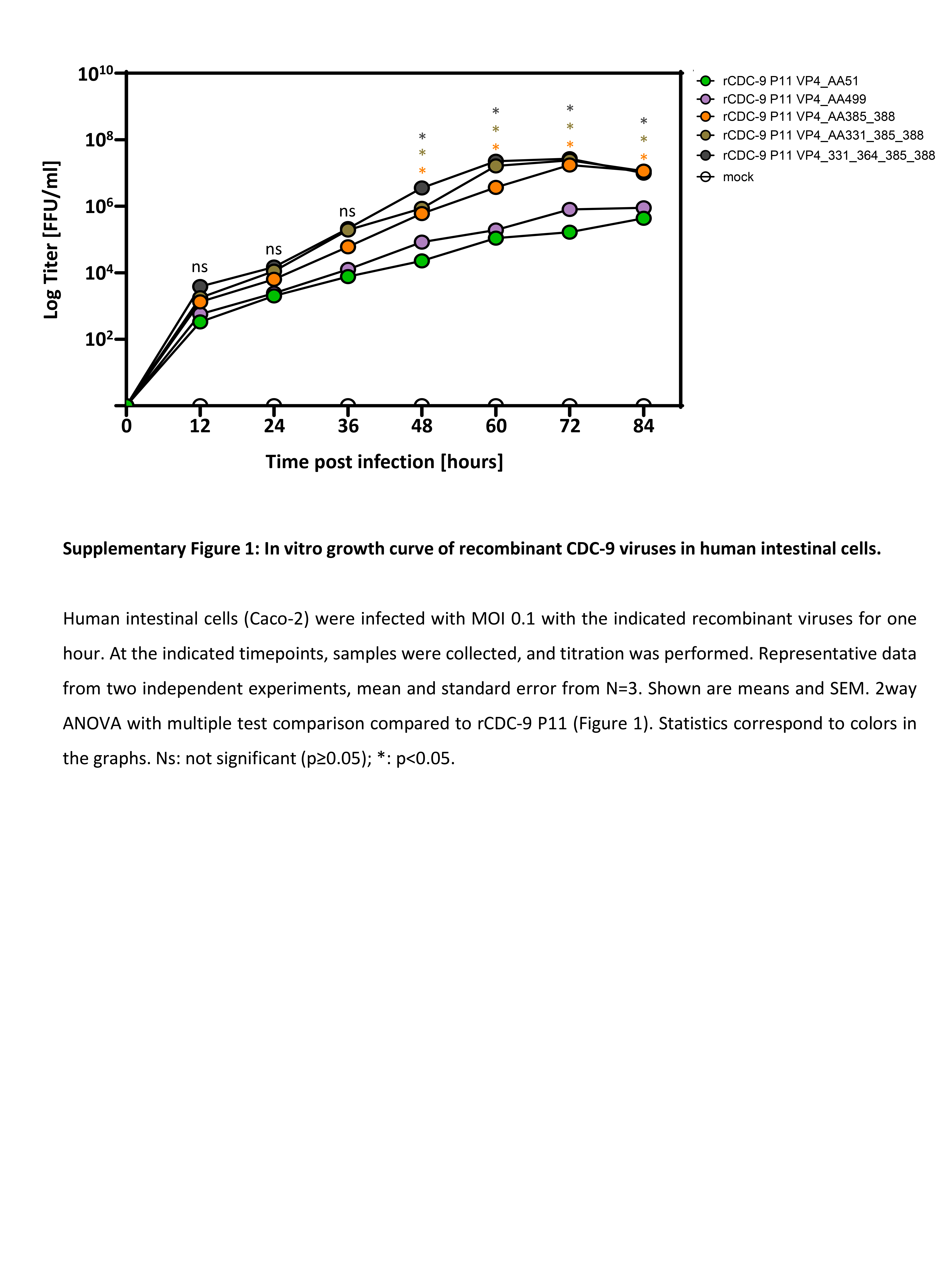

Supplement: Fig. S1 — In vitro growth curve of recombinant CDC-9 viruses in human intestinal cells. [file jvi.01067-25-s0001.tif]

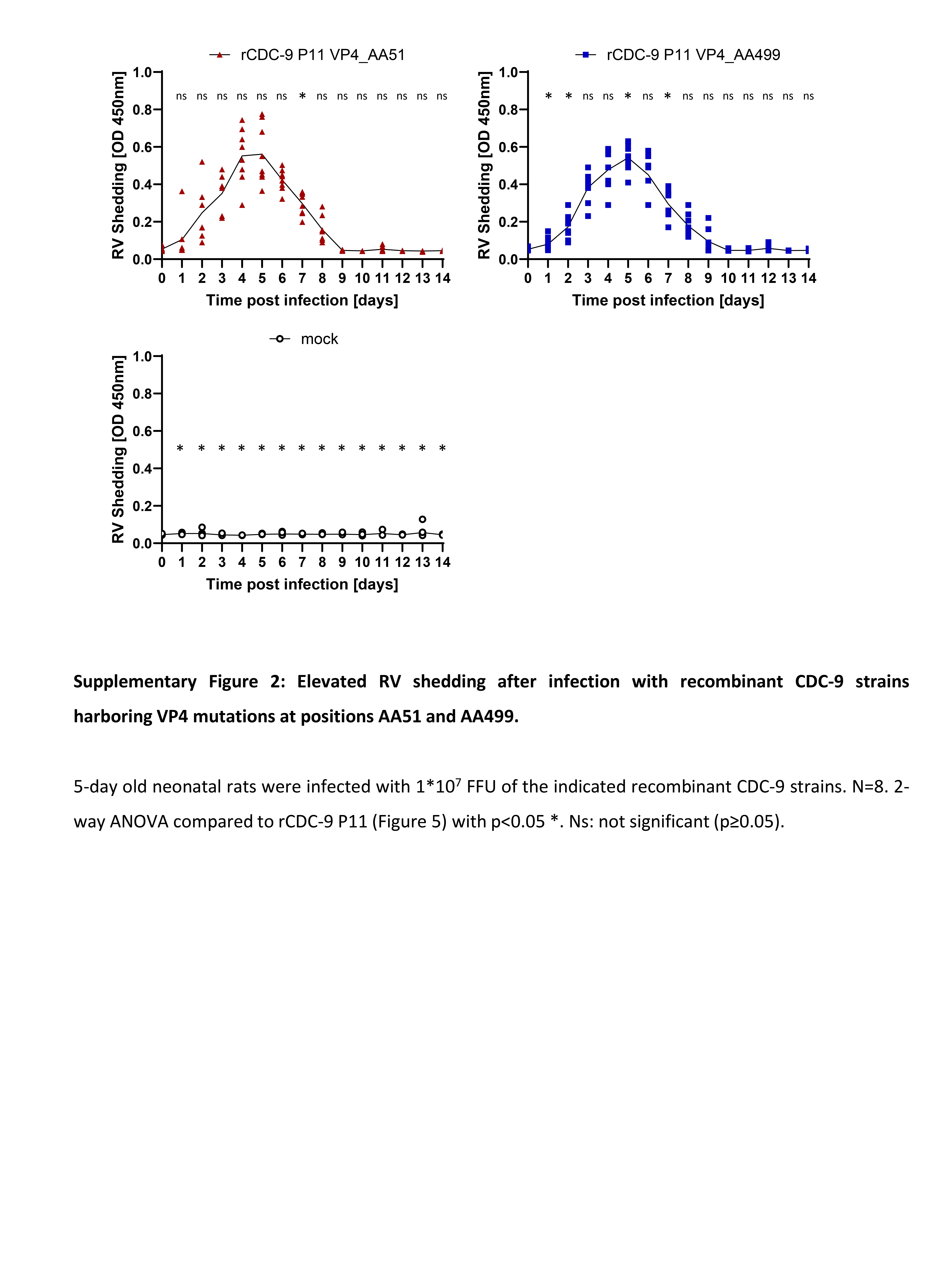

Supplement: Fig. S2 — Elevated RV shedding after infection with recombinant CDC-9 strains harboring VP4 mutations at positions AA51 and AA499. [file jvi.01067-25-s0002.tif]

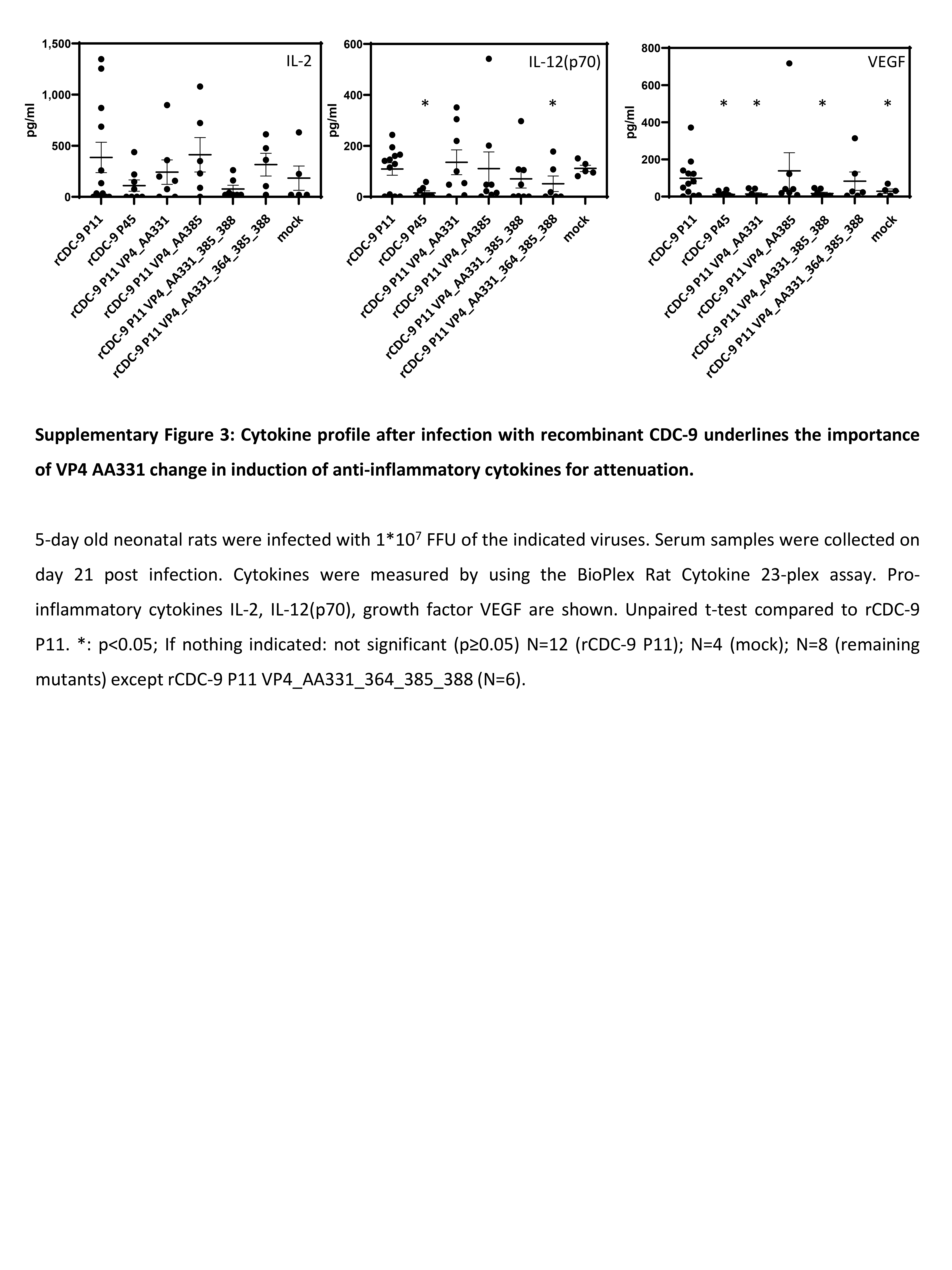

Supplement: Fig. S3 — Cytokine profile after infection with recombinant CDC-9 underlines the importance of VP4 AA331 change in induction of anti-inflammatory cytokines for attenuation. [file jvi.01067-25-s0003.tif]
